# Analysis of the Gut Bacterial Community of Wild Larvae of *Anastrepha fraterculus* sp. 1: Effect of Host Fruit, Environment, and Prominent Stable Associations of the Genera *Wolbachia*, *Tatumella*, and *Enterobacter*

**DOI:** 10.3389/fmicb.2022.822990

**Published:** 2022-03-10

**Authors:** Julieta Salgueiro, A. Laura Nussenbaum, Fabián H. Milla, Elias Asimakis, Lucía Goane, M. Josefina Ruiz, Guillermo E. Bachmann, María T. Vera, Panagiota Stathopoulou, Kostas Bourtzis, Ania T. Deutscher, Silvia B. Lanzavecchia, George Tsiamis, Diego F. Segura

**Affiliations:** ^1^Instituto de Genética “Ewald A. Favret” (INTA) – GV IABIMO (CONICET), Hurlingham, Argentina; ^2^Consejo Nacional de Investigaciones Científicas y Técnicas (CONICET), Buenos Aires, Argentina; ^3^Laboratory of Systems Microbiology and Applied Genomics, Department of Environmental Engineering, University of Patras, Agrinio, Greece; ^4^Facultad de Agronomía y Zootecnia, Universidad Nacional de Tucumán, San Miguel de Tucumán, Argentina; ^5^Insect Pest Control Laboratory, Joint FAO/IAEA Center of Nuclear Techniques in Food and Agriculture, Vienna, Austria; ^6^Biosecurity and Food Safety, NSW Department of Primary Industries, Elizabeth Macarthur Agricultural Institute (EMAI), Menangle, NSW, Australia

**Keywords:** microbiome, South American fruit fly, Tephritidae, sterile insect technique, next-generation sequencing, 16S rRNA

## Abstract

The genus *Anastrepha* (Diptera Tephritidae) includes some of the most important fruit fly pests in the Americas. Here, we studied the gut bacterial community of 3rd instar larvae of *Anastrepha fraterculus* sp. 1 through Next Generation Sequencing (lllumina) of the V3-V4 hypervariable region within the 16S rRNA gene. Gut bacterial communities were compared between host species (guava and peach), and geographical origins (Concordia and Horco Molle in Argentina) representing distinct ecological scenarios. In addition, we explored the effect of spatial scale by comparing the samples collected from different trees within each geographic origin and host species. We also addressed the effect of fruit size on bacterial diversity. The gut bacterial community was affected both by host species and geographic origin. At smaller spatial scales, the gut bacterial profile differed among trees of the same species and location at least in one host-location combination. There was no effect of fruit size on the larval gut bacteriome. Operational Taxonomic Units (OTUs) assigned to *Wolbachia*, *Tatumella* and *Enterobacter* were identified in all samples examined, which suggest potential, non-transient symbioses. Better knowledge on the larval gut bacteriome contributes valuable information to develop sustainable control strategies against *A. fraterculus* targeting key symbionts as the Achilles’ heel to control this important fruit fly pest.

## Introduction

The study of symbiotic relationships between insects and microbial organisms has received renewed interest as next-generation sequencing (NGS) tools become progressively available. Uncultured microbes that live in association with insects can now be studied by applying novel bioinformatic tools *in silico* (Nikolaki and Tsiamis, 2013; Knight et al., 2018). Recent studies in metabolic roles and host-microbiota associations reveal that symbionts have co-evolved with the host and are involved in essential physiological functions, thus modulating host fitness. This has changed the way insects are considered, from individuals to complex communities (Feldhaar, 2011; Douglas, 2015; Morris, 2018; Brown et al., 2020). Symbioses between insects and their microbiota also have practical implications for insect pest management, as acquired knowledge may lead to sustainable control methods (Qadri et al., 2020).

Although different types of microbes are hosted by insects, the gut bacteriome has received more attention in the last decades (Bourtzis and Miller, 2003; Gurung et al., 2019). Insect gut bacterial communities vary markedly from one species to the next, not only in the total abundance of bacteria that inhabit their gut [from 10^5^ bacteria in *Drosophila melanogaster* to ca. 10^9^ in the honey bee (Engel and Moran, 2013)], but also in terms of bacterial richness. Colman et al. (2012), based on the 16S rRNA gene sequence, found insect species guts harbored > 100 Operational Taxonomic Units (OTUs), whereas other insect groups, such as bees and wasps, harbored ca. 10 OTUs. Despite the differences in richness, extensive analyses of the insect gut bacteria reported a general trend to a low bacterial diversity in the insect gut (Colman et al., 2012; Jones et al., 2013). These symbiotic associations may have a variety of negative, positive, or neutral effects on the insect host. Furthermore, specific bacteria may have a different effect in insect hosts of different species (Bourtzis and Miller, 2003).

Family Tephritidae (Diptera) is distributed around the world and comprises approximately 5,000 species (White and Elson-Harris, 1992; Qin et al., 2015). Several Tephritidae species are known as fruit flies because larval development occurs inside fruit and are thus considered important quarantine pests worldwide (Bragard et al., 2020). On top of the direct damage to fruit, fruit fly pests impose restrictions to the access of potential markets in fruit fly free countries (Borges-Soto et al., 2019). Furthermore, niche expansion due to climatic warming (Bale et al., 2002; Lehmann et al., 2020) threatens the sustainability of areas free of fruit flies (Sutherst et al., 2000).

After NGS became available, the diversity and complexity of the interactions between bacteria and Tephritidae fruit flies started to be explored (Noman et al., 2020). Based on the species studied so far, fruit flies host diverse members of Enterobacteriaceae; *Enterobacter* being the most abundant genus (Raza et al., 2020). The gut bacterial community has been found to be affected by diet, host taxonomy and developmental stage (Aharon et al., 2013; Augustinos et al., 2019; Deutscher et al., 2019; Majumder et al., 2020; Noman et al., 2020). Recent studies in species of *Bactrocera* and *Ceratitis* suggest that geographic origin might also determine the composition of the gut microbiome (Liu et al., 2018; Koskinioti et al., 2019; De Cock et al., 2020). Even though most studies have been focused on adult, gut, bacteria in laboratory-reared flies (Deutscher et al., 2019) those that include other developmental stages report an effect of ontogeny on the microbial profiles of Tephritid flies (Estes, 2009; Yun et al., 2014; Andongma et al., 2019).

Even though the knowledge about bacterial symbionts in Tephritidae has increased over the past two decades, studies addressing their role are scarce (Augustinos et al., 2021). Potential roles of gut bacteria include insecticide resistance by degradation of pesticides (Vontas et al., 2011), nitrogen fixation (Behar et al., 2005; Bar-Shmuel et al., 2019), protein synthesis (Ben-Yosef et al., 2008) and amino-acids provisioning (Miyazaki et al., 1968). Improvement of nutritional status, derived from gut bacteria, has been studied in adults and indirectly linked to higher male sexual performance, increased flight ability, and starvation endurance (Ben-Yosef et al., 2008; Niyazi et al., 2009; Ben Ami et al., 2010; Gavriel et al., 2011; Augustinos et al., 2015; Noman et al., 2020). In *Bactrocera dorsalis*, the absence of gut bacteria affects, at least indirectly, the foraging behavior of adults, which could indicate that flies need to compensate specific nutrients provided by bacteria (Akami et al., 2019). In the case of larvae, Ventura et al. (2018) studied the gut bacteriome in four *Anastrepha* species and performed microbial metabolic predictions that suggested participation of bacteria in metabolic pathways related to membrane transport and metabolism of carbohydrates, amino acids, cofactors, and lipids. In a more direct approach, Zaada et al. (2019) demonstrated the fundamental role of gut bacteria in allowing *C. capitata* larvae to develop in unripe fruit.

The South American fruit fly, *Anastrepha fraterculus*, is one of the most important fruit fly pests in South America (Cladera et al., 2014). *Anastrepha fraterculus* is a complex, cryptic species, with at least eight distinct morphotypes (Hernández-Ortiz et al., 2012), which shows a high degree of sexual isolation (Vera et al., 2006; Cáceres et al., 2009; Segura et al., 2011; Abraham et al., 2014; Juárez et al., 2015). In Argentina, only the morphotype, Brazilian 1 or *A. fraterculus* sp. 1, has been reported (Hernández-Ortiz et al., 2012). The gut bacterial community of *A. fraterculus* sp. 1 has only been studied in the adult stage (Augustinos et al., 2019; Conte et al., 2019; Devescovi et al., 2019; Juárez et al., 2019; Salgueiro et al., 2020). The intimate association between fruit and fruit fly larvae envisions that fruit should influence their gut bacteria. In fact, a significant role of the host fruit on the gut bacteriome has been described for other fruit fly species (Behar et al., 2005; Thaochan et al., 2013; Deutscher et al., 2018; Ventura et al., 2018; Majumder et al., 2019; Zaada et al., 2019).

In the present study we aimed to characterize the gut bacterial community of *A. fraterculus* sp. 1 and to compare the gut bacteriome hosted by wild larvae that feed on two distinct host species. We included a native (*Psidium guajava*) and an exotic (*Prunus persica*) host species. Since endophytic bacteria are greatly affected by climatic conditions and location (Nair and Padmavathy, 2014), we also compared the gut bacteriome of larvae collected in two different ecosystems. Furthermore, we explored the effect of the spatial scale at a finer grain, by comparing the diversity in gut bacterial community among fruits collected from different trees within each geographic origin and host species. *Anastrepha fraterculus* females deposit a host marking pheromone (HMP) on the fruit surface that deter from egg laying in co-specific females (Prokopy et al., 1982; Liendo et al., 2020). Because HMPs efficacy is negatively correlated with the size of the fruit (Silva et al., 2012), larger fruits are expected to be infested by a larger number of females. This might lead to larger variability in the bacteria inoculated during oviposition (Behar et al., 2005; Zaada et al., 2019). This hypothesis was tested as part of the present study, comparing gut bacterial diversity among fruits of different size.

## Materials and Methods

### Sampling

*Anastrepha fraterculus* sp. 1 larvae were collected from infested peaches and guavas, two host species that exhibit heavy infestation by *A. fraterculus* sp. 1 (Schliserman et al., 2004; Segura et al., 2006).

For the same host fruit, diversity of gut bacterial profiles was analyzed at different spatial scales: (i) geographic origin; (ii) tree. Two geographic origins, that show different biotic and abiotic conditions, were considered: Horco Molle (26° 49′ 00′′ S, 65° 19′ 00′′ W) located to the northwest of Argentina, in Tucuman Province, and Concordia (31° 23′ 32′′ S, 58° 01′ 01′′ W) located to the northeast of Argentina, in Entre Rios Province. In Horco Molle, guava grow wild at the eastern edge of the Yungas rainforest, while peach trees are normally grown in backyards. Horco Molle is approximately 5 km from an area that is extensively used to produce lemons. The climate of the area is temperate-humid, with dry winters and rainy summers. The average annual temperature is 18°C, and the average annual rainfall is 1,330 mm. Concordia (C) belongs to the Pampeana region, characterized by a temperate climate. The annual mean temperature is 18.7°C, with 1,372.6 mm of precipitation (Ramos et al., 2018). Citrus, blueberry, and forestry plantations dominate the area.

Within each geographic origin and host species, six trees were randomly selected and five infested fruits, also randomly chosen, were collected per tree. In total, 120 samples were collected (two locations × two hosts × six trees × five fruits per tree). Infested fruits were individually weighed and then dissected. From each fruit, five larvae were extracted and taxonomically identified as *A. fraterculus* sp.1, through the morphology of their posterior spiracles, under a stereoscopic microscope (Olympus SZ30, 20X zoom) following Norrbom et al. (2012).

### Digestive Tracts Dissection and DNA Isolation

Wild, third instar larvae of *A. fraterculus* sp. 1 were surface sterilized, and subsequently dissected in a laminar flow hood, following procedures described in Salgueiro et al. (2020) for adult flies. Sterilization was carried by rinsing the larvae in a sequence of sterilized distilled water (sdw), sodium hypochlorite 0.05%, ethanol 70%, and sterile PBS 1X, for 1 min each. Mid and posterior gut were extracted with sterile dissecting forceps in PBS 1X under a stereoscopic microscope (Olympus SZ30, 40X zoom). For DNA extraction, guts from five larvae were pooled per sample to reduce inter-individual variability. DNA extraction was performed following Baruffi et al. (1995) with modifications related to the size of the sample, as it was reported previously (Salgueiro et al., 2020). The quantity and quality of the extracted DNA was measured in each sample by means of a NanoDrop 1000 (Thermo Fisher Scientific, Wilmington, NC, United States).

### Library Preparation and Illumina MiSeq Sequencing

A quantity of 50 ng of DNA per sample was used as template to generate amplicons corresponding to the V3-V4 hypervariable region of the bacterial 16S rRNA gene. A first round of PCR amplification was performed using KAPA HiFi HotStart PCR Kit (Kapa Biosystems) and MiSeq primers 341F and 805R (Klindworth et al., 2013). PCR products obtained were separated in a 1.2% w/v agarose gel electrophoresis to verify their size. The amplification products were visualized in Bio-Rad’s Gel Doc™ XR+ system. Positive PCR fragments were then purified from primers and primer dimers (Ntougias et al., 2016). The dried precipitates were suspended in 15 μl of sterile deionized water, diluted up to 10 ng/μl and used as template in a second round of PCR. In this step, indexed adapters were added to the ends of the 16S rDNA amplicons, as well as the Illumina adaptors. The combinatorial use of index primers resulted in unique samples that were pooled and sequenced on one Illumina MiSeq run. The resulting amplicons were cleaned-up by AMPure XP beads (Agencourt, United Kingdom) and diluted to 2.66 ng/μl. Finally, they were pooled equimolarly and mixed into an indexed library following the 16S-metagenomic library preparation guide 15044223-b (Illumina Inc, 2013). Massive Parallel Amplicon Sequencing was performed using an Illumina MiSeq sequencing platform by Macrogen. The entire dataset can be found at online repositories. All 16S rRNA gene sequences have been deposited in the NCBI (BioProject PRJNA779390).

### Data Analysis

The pre-processing of raw reads was carried out using USEARCH v10. Paired Fastq files were assembled by using algorithms implemented in USEARCH v10 using—fastq_mergepairs command with -fastq_maxdiffs, -fastq_pctid, -fastq_minmergelen, and -fastq_maxmergelen options set at default values. All reads were trimmed and filtered by quality using -fastq_filter, with the -fastq_maxee option set at 1.0 and unique sequences were identified by—fastx_uniques commands. All samples were clustered at increasing similarities of 97% using UPARSE-OTU algorithm (Edgar, 2013). Using this algorithm, chimera filtering and OTU clustering were carried out simultaneously. For the clustering, a minimum abundance (value = 2) was used discarding singletons. In addition, UNCROSS2 algorithm was run to detect and filter crosstalk (Edgar, 2018). For the OTU Table trimming, we defined 0.001 as the minimum frequency for an OTU. The OTU frequency was calculated as follows: (number of count reads for an OTU/total number of count reads)*100.

The taxonomy assignment was performed with QIIME2 (Bolyen et al., 2019) using a reference database (SILVA release 119; Quast et al., 2013). The OTUs identified as plastids were removed from the OTU table and from the fasta file. Finally, the commands—alignment mafft;—phylogeny fasttree were run in QIIME2 to build the phylogenetical tree.

A heatmap was obtained with WPS Spreadsheets (Kingsoft, 2020). For each of the four groups that resulted from combining host species and location (PC: peach from Concordia; PH: peach from Horco Molle; GC: guava from Concordia; GH: guava from Horco Molle), mean fruit weight was calculated and used as reference value: fruit that weighed more than the average were classified as “AA” (above average), while fruits that weighed less than the average were assigned to group “BA” (below average).

A Venn diagram was calculated to analyze shared and unique OTUs among the combination of host species and locations (PC, PH, GC, GH) by means of the Bioinformatics and Evolutionary Genomics online platform (VIB - Ghent University, n.d.).

Diversity estimates including observed OTUs and Good’s Coverage were calculated using final count data. Alpha diversity indexes included richness (Chao1), diversity (Simpson and Shannon), dominance (Berger– Parker) and evenness (Pielou) which reflect the diversity of individual samples. These indexes were calculated using “vegan” R package (Oksanen et al., 2019) and were plotted with “ggplot2” R Package (Wickham, 2016).

Phylogenetic diversity (Faith index) was estimated using “Picante” package in R (Kembel et al., 2010). Alpha diversity indexes were compared by pairwise Kruskal–Wallis tests in R (R Core Team, 2020).

Beta diversity was analyzed using Generalized UniFrac distance (Chen et al., 2012) and visualized *via* Non-metric Multidimensional Scaling (NMDS) plot using the RHEA pipeline in R (Lagkouvardos et al., 2017). A permutational multivariate analysis of variance (PERMANOVA; Anderson, 2001) was performed using “adonis” function from “vegan” R package (Oksanen et al., 2019). The Bonferroni–Hochberg method was used to correct for multiple PERMANOVA testing.

The mean proportion of sequences within each OTU were compared between groups using STAMP (Parks et al., 2014). The plots also show the difference in mean proportions for each pair of comparisons and a *p*-value indicating if the mean proportion is equal for a given pair calculated by Welch’s T- test (Bluman, 2009) and corrected by Storey’s FDR (Storey et al., 2004).

## Results

### Overall Data Analysis

After the trimming process, 5,147,567 high quality reads were obtained from the bacterial community of 120 larval gut samples of *A. fraterculus* sp. 1. After finding the unique sequences and performing the corresponding clustering, 703 OTUs were identified, and 2,059 chimeras were discarded. The normalized OTU table was cleaned up identifying potential “cross-talk” and trimming the table, deleting 659 OTUs. Once the taxonomy alignment was performed in QIIME2, 4 OTUs were identified as organelles and consequently discarded. This procedure led to a set of 40 bacterial OTUs clustered at 97% sequence similarity (Supplementary Table 1).

**FIGURE 1:**
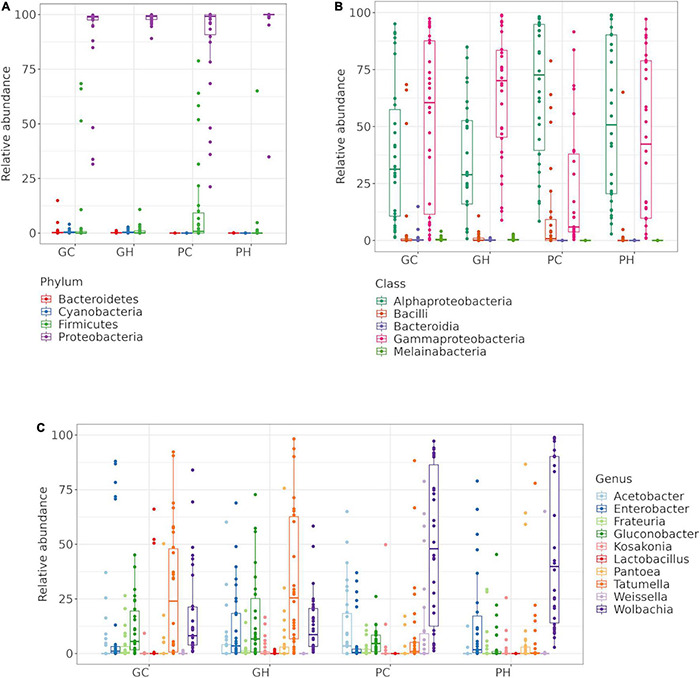
Larval gut bacteria of *Anastrepha fraterculus* sp. 1. Relative abundance of the ten main OTUs identified. Taxonomic identification at: **(A)** Phylum, **(B)** Class and **(C)** Genus.

We found OTUs belonging to four bacterial phyla, five classes, and 13 orders in the gut bacterial of *A. fraterculus* sp. larvae 1 (Figure 1 and Supplementary Tables 1, 2). Two of the 40 OTUs could not be assigned taxonomy under order level (OTU18 and OTU319) and were, therefore, registered as unknown Enterobacterales. Likewise, three OTUs could only be assigned to family level: Micropepsaceae-OTU22, Chitinophagaceae-OTU40, Orbaceae-OTU42.

One OTU, OTU17, was assigned to phylum Cyanobacteria, class Melainabacteria, order Oscuribacterales, being a new unknown Cyanobacterium. Due to the phylogenetic relationship between chloroplasts and Cyanobacteria and the fact that the gut bacteriome analyzed belongs to endophytic larvae, it was rechecked to avoid any bias. Thereby, the OTU assigned to Cyanobacteria was aligned by BLAST with Nucleotide collection (nr/nt) database filtering by “Cyanobacteria” (taxid: 1117), obtaining “Uncultured Cyanobacterium” (ID: KU667126.1) the lowest e-value (0,0) and 100% of identity.

Two OTUs, OTU40 and OTU26, were assigned to class Bacteroidia from phylum Bacteroidota (formely Bacteroidetes), identified as unk_Chitinophagaceae (OTU40) and Chishuiella (OTU26). Within Firmicutes, only class Bacilli was identified. Conversely, two classes of Proteobacteria, Alphaproteobacteria and Gammaproteobacteria, were found to be the most abundant and diverse represented phyla. Indeed, at family level, we found Enterobacteriaceae, which belongs to Gammaproteobacteria, as the most abundant family, followed by Anaplasmataceae in samples from peaches and Acetobacteraceae in samples from guava (Supplementary Table 1).

**FIGURE 2:**
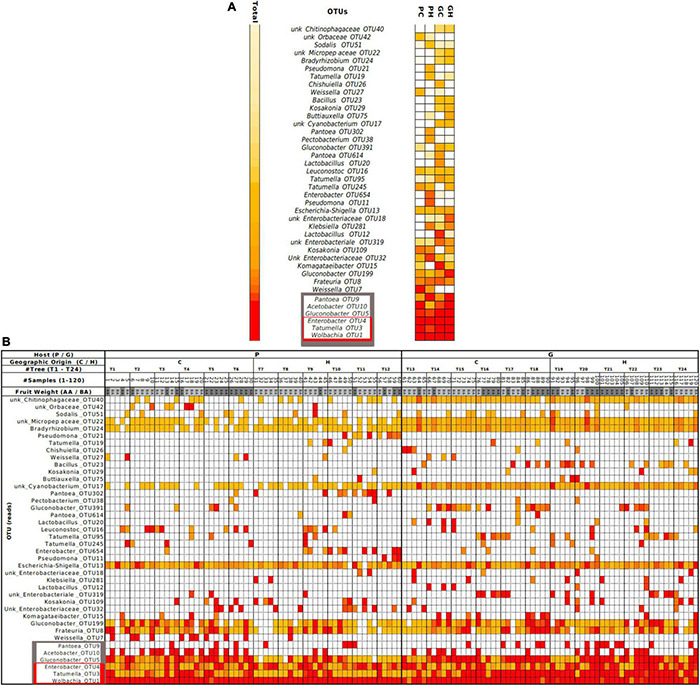
The heatmap presents each of the OTUs identified in the gut bacteriome of wild *Anastrepha fraterculus* larvae, ordered from top to bottom according to total counts. **(A)** Summary of counts grouped by host and origin (PC, peach sampled in Concordia; PH, peach sampled in Horco Molle; GC, guava sampled in Concordia; GH, guava sampled in Horco Molle). **(B)** Complete dataset visualization ordered by total reads (see data in Supplementary Table 2). Color assignment: red: 90th percentile; orange: 50th percentile; white: 0 read. AA, fruit weight above mean weight; BA, fruit weight below mean weight (estimated separately for each host fruit species).

Relative abundance at genus level revealed 10 main genera found across GC, GH, PC, and PH: *Acetobacter, Enterobacter, Frateuria, Gluconobacter, Kosakonia, Lactobacillus, Pantoea, Tatumella, Weissella* and *Wolbachia* (Figure 1C). At the OTU level, the heatmap (Figure 2—gray box) highlights the relative abundance of the six most dominant OTUs: Pantoea-OTU9, Acetobacter-OTU10, Gluconobacter-OTU5, Enterobacter-OTU4, Tatumella-OTU3, and Wolbachia-OTU1. Conversely, some OTUs like unk_Chitinophagaceae-OTU40 and unk_Cyanobacterium-OTU17, show a medium to low relative abundance but were detected in many samples (Figure 2 and Supplementary Table 2). Escherichia-Shigella-OTU13, with an intermediate relative number of reads, was detected in almost all samples (Figure 2 and Supplementary Table 1).

**FIGURE 3:**
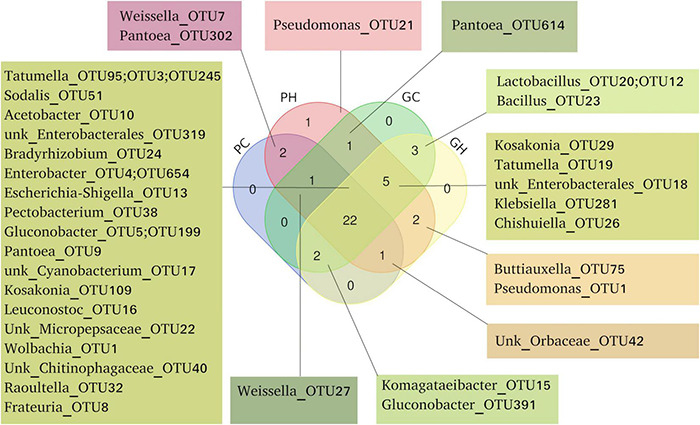
Shared and unique OTUs found in the gut microbiome of *Anastrepha fraterculus* larvae. Venn diagram showing OTUs shared across all four origins and hosts combinations (PC, peach sampled in Concordia; PH, peach sampled in Horco Molle; GC, guava sampled in Concordia; GH, guava sampled in Horco Molle), three and two of them. OTUs are presented in detail, including number and taxonomic identification according to Silva database.

Figure 3 represents exclusive and shared OTUs from each sample as a Venn diagram. Some OTUs were exclusively associated with the host species, as Weissella-OTU7 and Pantoea-OTU32 from peach, and Lactobacillus-OTU20, OTU12 and Bacillus-OTU23 from guava. Some OTUs were exclusively associated with one geographic origin. Guavas and peaches from Horco Molle (GH and PH) showed two shared OTUs, Buttiauxella-OTU75 and Pseudomonas-OTU11, which were not detected in the rest of the samples. Conversely, Pseudomonas-OTU21, was detected exclusively in PH samples (Figure 3). Regardless of host fruit species and location, we found 22 shared OTUs (Figure 3 and Supplementary Table 1). Eleven of these OTUs were present in more than 50% of the total samples (120).

### Influence of Host Fruit

**FIGURE 4:**
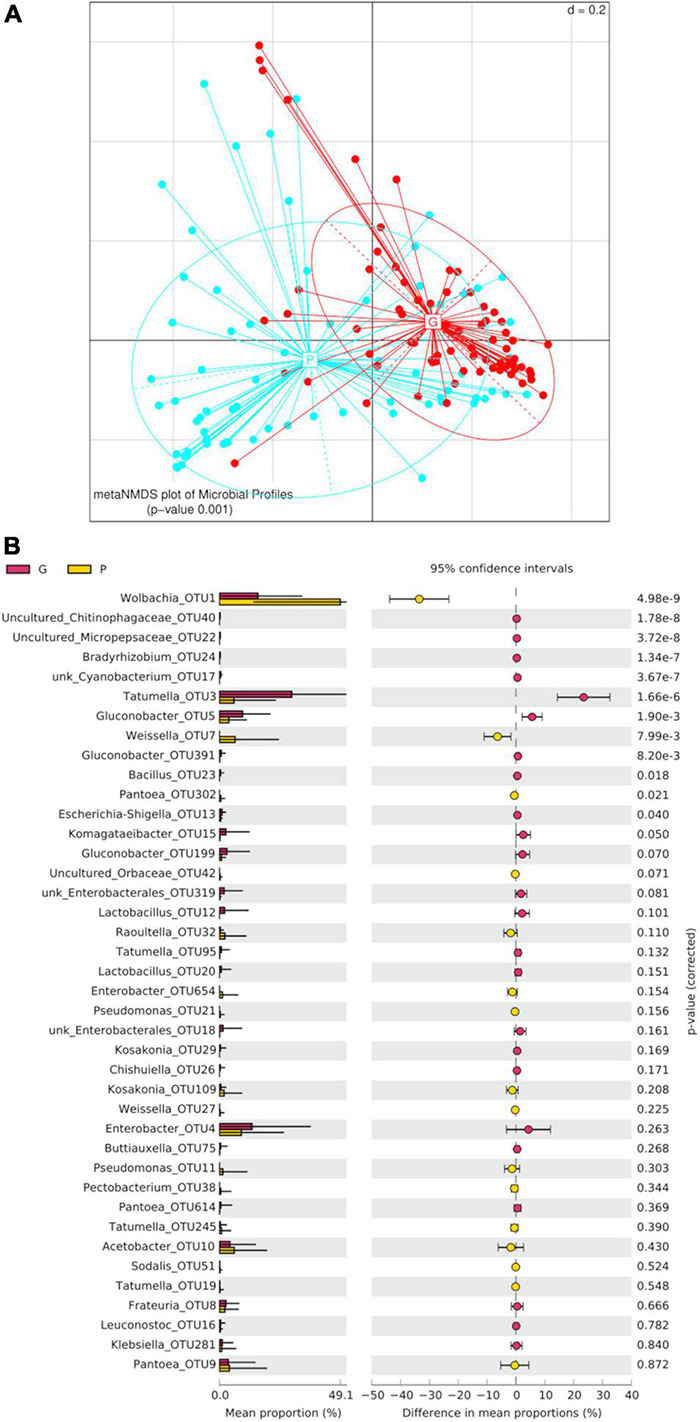
Host fruit effect on the larval gut bacteriome of wild *Anastrepha fraterculus*. **(A)** Meta Non-metric Multidimensional scaling (meta NMDS) plot representing sample groups according to the host fruit where larvae originated: guava (G) or peach (P). Significance *p*-value from PERMANOVA analysis; *d* = 0.2. **(B)** Host fruit effect on OTUs relative abundance mean: the plot presents the mean proportion of reads for each OTU to the left (bars show mean value and standard error) the difference in mean proportions for each pair of comparisons (as well as its associated *p*-value according to Welch’s *T*-test).

**FIGURE 5:**
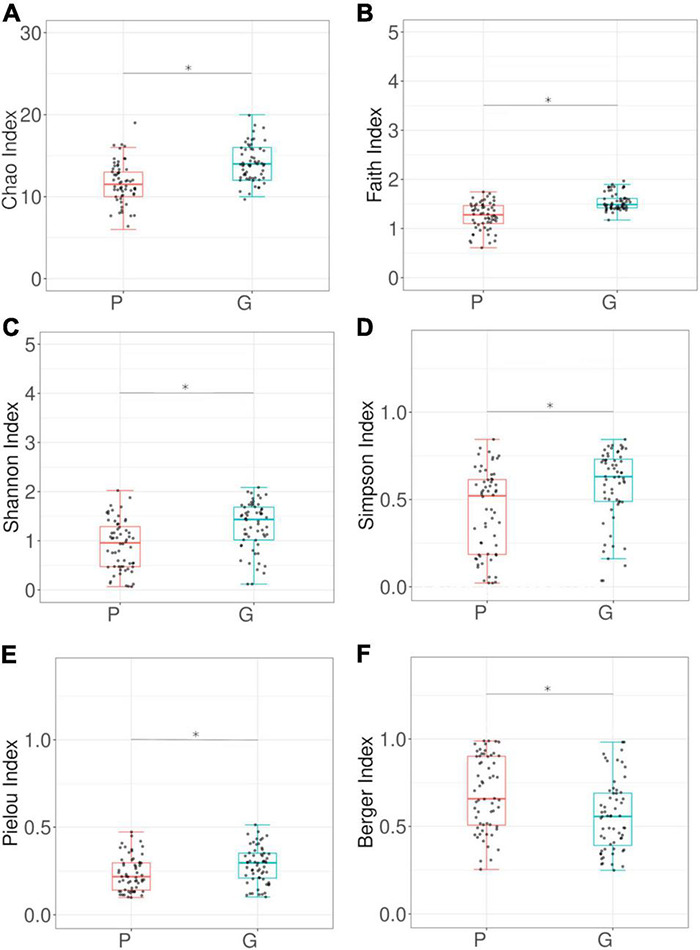
α-diversity analyses of larval gut bacterial community associated to wild *Anastrepha fraterculus*. The figure presents the comparison between larvae recovered from different host species, guava (G) and peach (P). **(A)** Chao index; **(B)** Faith index; **(C)** Shannon index; **(D)** Simpson Index; **(E)** Pielou Index; **(F)** Berger Index. Dots indicate observed values and box plots depict means and standard deviation. Kruskal–Wallis Rank Sum test *p*-values are plotted for each paired comparison.

Beta-diversity analysis of the bacterial community shows that the profiles differed significantly between larvae from different hosts (PERMANOVA *p*-value 0.001) (Figure 4A). Alpha-diversity indices (Figure 5) reveal that the gut bacterial community of *A. fraterculus* sp. 1 larvae sampled from guava presents significantly higher richness, phylogenetic diversity (Faith index), evenness (Pielou index), and Shannon and Simpson diversity than samples from peach. Congruently, peach presents higher levels of dominance estimated by means of the Berger-Parker index (Supplementary Figure 1). OTUs abundance per host fruit is plotted in Figure 4B. Significant higher values in mean proportions were found for Wolbachia-OTU1, Weissella-OTU7 and Pantoea-OTU302 from peach samples in comparison with guava samples. Conversely, Tatumella-OTU3, Gluconobacter-OTU5, Bacillus-OTU23 and Escherichia-Shigella-OTU13 were significantly higher in larvae recovered from guava than samples from peach. Similarly, Chitinophagaceae-OTU40, Micropepsaceae-OTU22, Bradyrhizobium-OTU24, Cyanobacterium-OTU17 and Gluconobacter-OTU391 were significantly more abundant in samples from guava than in samples from peach, despite their low percentage of reads (Supplementary Table 2 and Figure 2B).

### Influence of Origin at Different Scales Within Each Host Fruit

**FIGURE 6:**
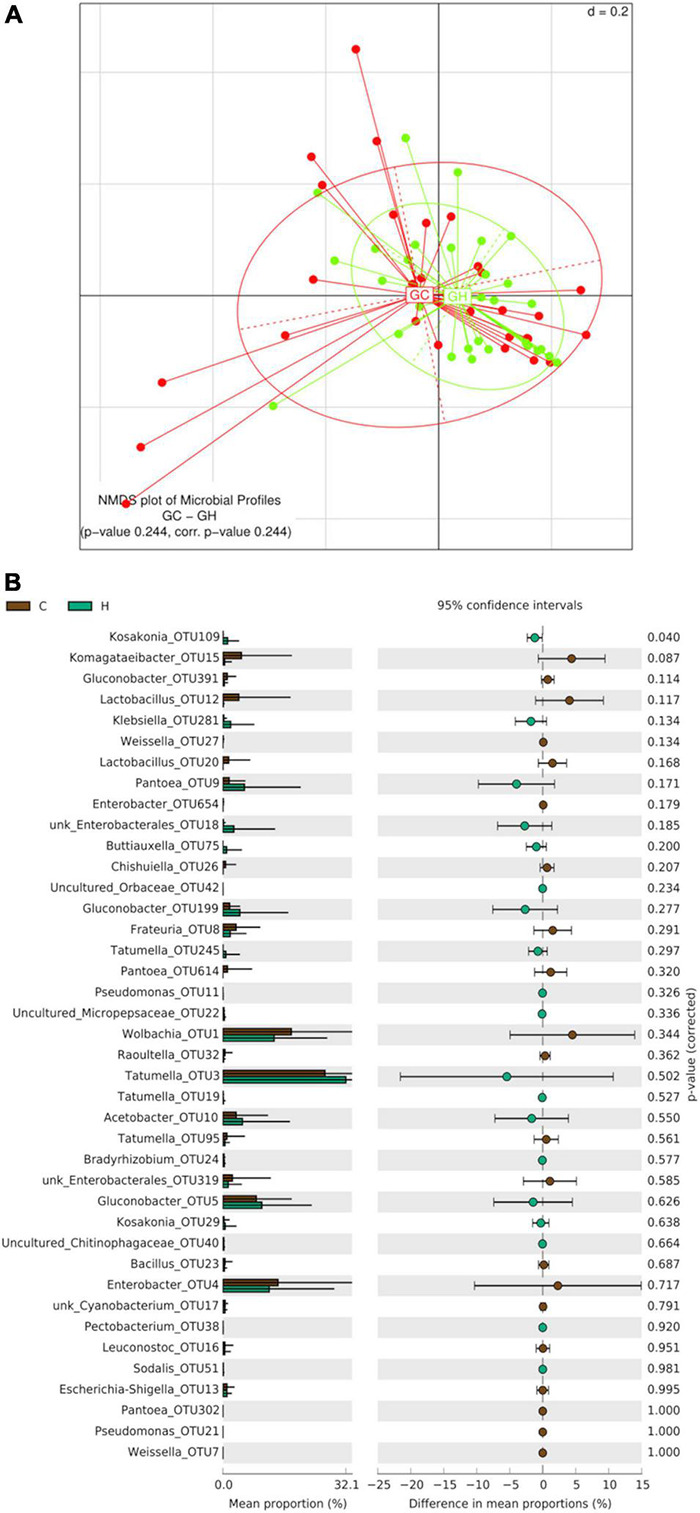
Effect of geographic origin on the larval gut bacteriome of wild *Anastrepha fraterculus* sampled in guava fruit. **(A)** Meta Non-metric Multidimensional scaling (meta NMDS) plot representing sample groups according to the area where fruit was collected: C (Concordia, Entre Ríos) or H (Horco Molle, Tucumán). Not significant *p*-value from PERMANOVA analysis; *d* = 0.2. **(B)** Origin effect on OTUs relative abundance mean: the plot presents the mean proportion of reads for each OTU to the left (bars show mean value and standard error) the difference in mean proportions for each pair of comparisons (as well as its associated *p*-value according to Welch’s *T*-test).

Beta-diversity analyses of guava samples did not reveal significant differences between the gut bacterial profile of larvae collected in Horco Molle and those collected in Concordia (PERMANOVA *p*-value 0.244) (Figure 6A). The analysis of alpha-diversity comparing guava samples between origins did not reveal significant differences in any of the indices studied here (Kruskal–Wallis Rank Sum test) (Supplementary Figure 1). However, the comparison of OTUs mean proportion between Horco Molle and Concordia, considering only larvae recovered from guava, revealed that Kosakonia-OTU109 is significantly more abundant in samples from Horco Molle (*p*-value 0.040) (Figure 6B). Conversely, Komagataeibacter-OTU15 was found in higher abundance in larval guts collected from guavas in Concordia. Tatumella-OTU3, Wolbachia-OTU1 and Enterobacter-OTU654 showed high relative abundance in all guava samples, with no difference between origins.

**FIGURE 7:**
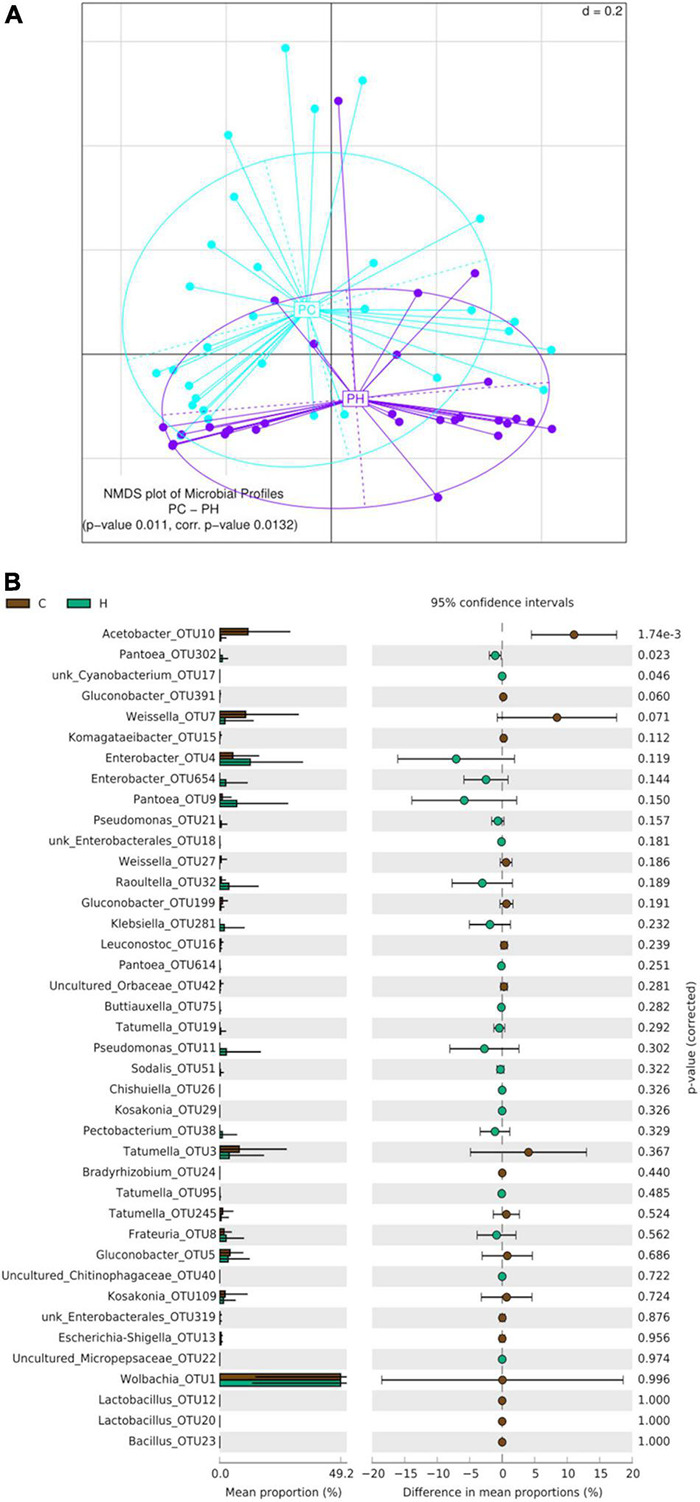
Effect of geographic origin on the larval gut bacteriome of wild *Anastrepha fraterculus* sampled in peaches. **(A)** Meta Non-metric Multidimensional scaling (meta NMDS) plot representing sample groups according to the area where fruit was collected: C (Concordia, Entre Ríos) or H (Horco Molle, Tucumán). Significant *p*-value from PERMANOVA analysis; *d* = 0.2. **(B)** Origin effect on OTUs relative abundance mean: the plot presents the mean proportion of reads for each OTU to the left (bars show mean value and standard error) the difference in mean proportions for each pair of comparisons (as well as its associated *p*-value according to Welch’s *T*-test).

When beta-diversity was analyzed considering exclusively the samples from peach, two groups of samples, one corresponding to Concordia and the second to Horco Molle, were detected as the result of generalized UniFrac distances processing (PERMANOVA corr. *p*-value 0.0132) (Figure 7A). Moreover, OTUs mean proportion comparison showed that in peaches from Concordia, the relative abundance of Acetobacter-OTU10 is higher than in Horco Molle (Figure 7B). On the contrary, unk_Cyanobacterium-OTU17 and Pantoea-OTU302, despite the low quantity of reads, were found in higher abundance in Horco Molle compared to Concordia. The analyses of α-diversity comparing samples of peach between origins did not reveal significant differences (Kruskal–Wallis Rank Sum test, Supplementary Figure 2).

### Tree and Fruit Weight Effect Over Larval Gut Bacterial Community

**FIGURE 8:**
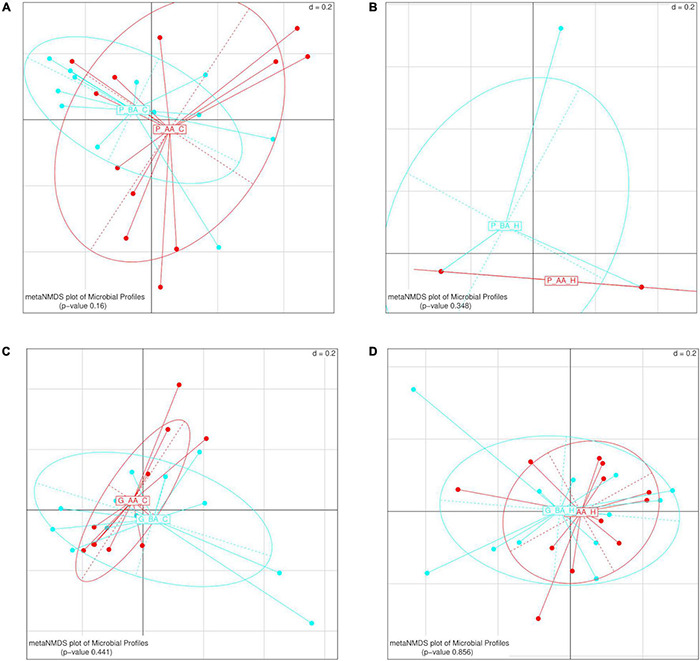
β-diversity analyses of the gut bacterial profile of wild *Anastrepha fraterculus* larvae recovered from fruits of different weight, within the same host fruit species and geographic origin. The effect of weight was plotted as Meta Non-metric Multidimensional scaling (meta NMDS) graph. **(A)** Peaches sampled in Concordia; **(B)** peaches sampled in Horco Molle; **(C)** guavas sampled in Concordia; **(D)** guavas sampled in Horco Molle. The *p*-values correspond to PERMANOVA tests. AA, fruits whose weight was above the mean weight across all sampled fruits of the same species; AB: AA, fruits whose weight was below the mean weight across all sampled fruits of the same species.

When the gut diversity was compared between samples of different trees, for each host species and location (PC, PH, GC, and GH), significant differences were found only for guavas sampled in Concordia (PERMANOVA *p*-value 0.004) (Table 1). Furthermore, we analyzed whether the weight of fruit affected the diversity of the gut bacteriome, considering each host and location separately. No significant differences were detected between the gut bacterial community of larvae recovered from fruits that were below or above the average weight, for any of the combinations of hosts and locations (Figure 8).

**TABLE 1:** β-diversity analyses comparing the gut bacterial profile of wild *Anastrepha fraterculus* larvae collected from different trees from the same host fruit species and geographic origin.

Comparison	*P*-value	Significance
PC (T1-T6)	0.276	NS
PH (T7-T12)	0.191	NS
GC (T13-T18)	0.004	S
GH (T19-T24)	0.105	NS

*The p-values correspond to PERMANOVA tests.*

## Discussion

The bacterial community associated to *A. fraterculus* sp. 1 larval gut was analyzed by 16S rRNA gene amplicon sequencing. We identified 40 bacterial OTUs belonging to four phyla. Diversity analyses provided evidence for a strong effect of the host fruit species, with higher bacterial diversity in the native (guava) than the exotic (peach) host. Larvae sampled from peaches showed a significant effect of the environment. Within each combination of host and location, we found no significant differences between fruits from different trees, except for guavas in Concordia. No effects of fruit size on the gut bacterial community were detected.

We found four bacterial phyla inhabiting the gut of *A. fraterculus* sp. 1 larvae: Bacteroidota, Cyanobacteria, Firmicutes and Proteobacteria. The most abundant and diverse phylum was Proteobacteria, in agreement with results previously reported for *A. fraterculus* sp. 1 adults (Augustinos et al., 2019; Juárez et al., 2019; Salgueiro et al., 2020). Müller (2013) found that the larval gut bacteriome of a Brazilian wild population of *A. fraterculus* was dominated by Actinobacteria. In this case, the author targeted a different region of the 16S rRNA gene, which could partially explain the differences; however, the fact that the morphotype of *A. fraterculus* studied is not indicated by Müller (2013) together with environmental variation, such as diet, might contribute to the differences observed with our study as well as with previous results (Augustinos et al., 2019; Salgueiro et al., 2020). For other *Anastrepha* species, a study carried out based on 454 pyrosequencing analysis, reported that the gut of larvae was dominated by *Escherichia* in *A. striata, A. ludens* and *A. obliqua*, and by *Raoultella* in *A. serpentina* (Ventura et al., 2018). In *A. fraterculus* sp. 1 larvae studied in the present work, *Escherichia-Shigella* and *Raoultella* represent 0.771 and 1.353% of the total reads, respectively. In the light of our findings and considering the effect of the host fruit on the larval gut bacteriome, the differences in bacterial abundance between the *Anastrepha* species studied by Ventura et al. (2018) and *A. fraterculus* sp. 1 could be mainly due to the host fruit sampled, that were different in these studies, except for *A. striata* which was recovered from guava. However, other factors, such as fly species or geographic origin, cannot be rule out. Actually, the fact that *A. striata* and *A. fraterculus sp.1* were both recovered from guava suggests that even though the host fruit seems like the most important factor shaping the gut bacteriome of *Anastrepha* larvae, other factors, can also play a significant role.

Regardless of the fruit (guava or peach) or the sampling area (Horco Molle or Concordia), we found 22 shared OTUs and, within them, a group of 10 OTUs were present in more than 50% of the samples. Most of these OTUs belong to genera that were already described in the gut of Tephritidae fruit flies, such as *Wolbachia, Tatumella, Enterobacter, Gluconobacter* and *Bradyrhizobium*. Even though these groups have been described as symbionts of fruit flies, in most cases their role has not been addressed and only suggested (e.g., nitrogen fixation by *Bradyrhizobium*) (Noman et al., 2020). However, the high representation of some bacterial taxonomic groups suggests they might have an important role in the physiology of their hosts, as was suggested for *Acetobacter tropicalis* in *Bactrocera oleae* (Kounatidis et al., 2009). In our case, we found *Wolbachia, Tatumella* (OTU3) and *Enterobacter* (OTU4) present in all gut samples of *A. fraterculus* sp. 1 larvae. A few highly represented taxa were not yet reported in Tephritidae, including *Frateuria, Escherichia-Shigella*, unknown Cyanobacterium and unk_Micropepsaceae. Interestingly, unk_Cyanobacterium-OTU17 belongs to Melainabacteria, a group that was proposed as a candidate phylum sibling to Cyanobacteria (Di Rienzi et al., 2013). The present work raises the question of whether Melainabacteria may also inhabit the gut of other Tephritidae species and has been ignored in previous reports, erroneously being dismissed as having chloroplast origin.

The taxonomy assignment detected potential new taxa that were not previously reported in *Anastrepha*. Five taxa were placed in distinct phylogenetic positions, showing lower than 97% similarity to any currently known 16S rRNA gene sequence. These taxa require further characterization through a metagenomic approach since they could potentially be members of novel bacterial species.

Wolbachia-OTU1 was found in all samples, in agreement with Müller (2013) for an unknown morphotype of *A. fraterculus* This bacterium has been found in approximately two thirds of Tephritidae fruit fly species studied so far (Mateos et al., 2020) *Wolbachia* was not detected in larvae of *A. serpentina*, *A. striata*, or *A. obliqua* by Ventura et al. (2018), but Gallo-Franco and Toro-Perea (2020) reported this bacterium as dominant in the gut of *A. obliqua* larvae. Recent evidence suggests that *Wolbachia* might have an indirect role during immature stages, particularly conditioning the abundance of *Enterobacter* in *A. obliqua* (Gallo-Franco and Toro-Perea, 2020). Bacterial interactions with *Wolbachia* may affect the gut bacteriome, such us the mutual exclusion of *Asaia* and *Wolbachia* in the reproductive organs of mosquitoes (Rossi et al., 2015). Crosstalk studies involving *Wolbachia* and other gut bacterial taxa will surely shed light on the role of this reproductive parasite during the larval stage.

Based on our results we propose two OTUs, besides Wolbachia-OTU1, as potential non-transient symbionts of *A. fraterculus* sp. 1: Enterobacter-OTU4 and Tatumella-OTU3. Regardless of the host fruit and the geographic origin, both OTUs, as well as *Wolbachia*, were detected in every single sample analyzed. *Enterobacter* has been detected in most Tephritidae fruit flies studied so far (Raza et al., 2020). *Tatumella*, on the other hand, has only been reported for *Bactrocera oleae* (Blow et al., 2020). According to Ventura et al. (2018), species with a wider host range may have more diverse bacterial communities than species with a narrow host range. In this line, it is interesting such a stable association with *Tatumella* in two species with such different host range, from highly polyphagous as *A. fraterculus* to the monophagous *B. oleae*. *Tatumella* was highly represented with four OTUs identified as part of this genus (OTUs 3, 19, 245, and 45). These findings foster studies on the localization and potential role of this bacterial group in *A. fraterculus* sp. 1 larvae.

Larvae collected from guavas and peaches showed different bacterial profiles. The gut community in larvae collected from guava showed higher richness, phylogenetic diversity, equity, and lower dominance than larvae collected from peaches. This pattern could be related with a longer time of co-evolution between *A. fraterculus* and guava, compared to peach, which is an exotic host fruit in South America. Further studies including a wide range of host species will aid to explore this hypothesis.

Regarding OTUs composition, we found that Tatumella-OTU3 and Gluconobacter-OTU5 were in significantly higher density levels in larvae from guava than larvae from peach, as well as other groups with lower percent of reads (e.g., Chitinophagaceae-OTU40, Micropepsaceae-OTU22, Bradyrhizobium-OTU24, Cyanobacterium-OTU17 and Gluconobacter-OTU391). The difference in bacterial groups could be associated with the suggested roles for some of these taxa (nitrogen fixation by *Bradyrhizobium* and *Cyanobacterium*, and polysaccharide degradation by *Gluconobacter* and Chitinophagaceae) which may be more advantageous in one of the two hosts. Majumder et al. (2019) showed that the bacterial community of *B. tryoni* larvae is also related with the bacterial community of the different host fruits where they develop, which might explain, at least in part, the differences between larvae recovered from different hosts in our study. Further work on nutrient content of each host species, bacterial community of the fruits, and larval nutritional needs may contribute to understanding the role of these bacteria to *A. fraterculus* sp. 1 development.

Some OTUs appear to be associated exclusively with one of the hosts evaluated. Weisella-OTU7 and Pantoea-OTU302 were only found in samples from peaches. Conversely, *Bacillus* and *Lactobacillus*, which belong to acetic acid bacteria (AAB), are characterized by inhabiting or even by generating low pH microenvironment and producing polysaccharidic matrices involved in gut protection (Crotti et al., 2010), were detected exclusively in guava samples.

The effect of the spatial scale on the gut bacterial profile of wild *A. fraterculus* sp. 1 larvae was studied at two levels: geographic origin and tree. Interestingly, some OTUs were present only in one origin, like Buttiauxella-OTU75, Pseudomonas-OTU11 and Pseudomonas-OTU21 (only detected in Horco Molle). Geographic origin had no effect on alpha diversity indices in guava or peach samples, but beta-diversity analysis showed significant differences between peach samples from Concordia and Horco Molle. This could result from differences between the two origins in terms of biotic as well as abiotic factors. Among abiotic factors, Feldhaar (2011) proposed that temperature can affect the abundance of bacteria within the host or their efficiency of transmission to the offspring. Horco Molle and Concordia have different temperature regimes, which could explain the effect of host location. In addition, the two areas belong to two distinct biographic regions, with a distinct fauna, which might translate in differences in the community of insects associated to the sampled fruits. We have no clear explanation as to why the gut bacterial community of larvae from peaches differ between origins, whereas larvae from guava showed no apparent effect of the origin. The fact that guava is a native host of *A. fraterculus* sp. 1 and environmental bacteria might have co-evolved and formed more stable associations with this insect species might explain this result. However, this explanation is highly speculative at this point and requires experimental documentation.

Regarding the smallest spatial scale (i.e., effect of the tree), the larval gut bacterial profile differed significantly between trees only in guavas from Concordia. In this area, trees were more distant among each other than in the rest of the groups. Even if these trees were exposed to similar environmental factors (temperature, precipitations, type of soil, etc.), our findings suggest that the surrounding environment significantly affected the host trees where larvae were collected. Recently, Yong et al. (2019) showed significant relationship between gut bacterial profile and the geographical region of *Zeugodacus cucurbitae.* To our knowledge, this is the first study reporting an effect of the spatial scale at this level of analyses involving region, host species and tree location for Tephritidae fruit flies.

Under the hypothesis that larger fruit have higher chances of being infested by more than one female, we would have expected larger variability in the bacteria inoculated during oviposition (Behar et al., 2008; Zaada et al., 2019). However, our results do not support this prediction, as fruit weight has no significant impact on the gut bacterial community. It could be argued that guavas and peaches are among the most infested hosts, reaching levels of bacterial diversity too high to detect an effect of host fruit size. Direct experiments testing the effect of single vs. multiple oviposition, in relation with the fruit size, might help to understand the extent to which gut bacterial diversity is explained by multiple females laying eggs in the same fruit.

Our findings indicate that the gut bacterial community of *A. fraterculus* sp. 1 larvae presents 22 common OTUs that may be involved in key functions during this stage. At the same time, this community is determined, at least to some extent, by the host fruit and by the geographic location of the fruit, and in some cases even by the tree where the larvae develop. Potential non-transient bacteria were also identified, including *Tatumella* (not previously reported in *A. fraterculus* sp. 1), *Enterobacter* and *Wolbachia*. It is important to note that our sampling was focused on third instar larvae. A recent study suggests that the gut bacteriome changes across larval stages in *Bactrocera minax* (Yao et al., 2019), thus future studies should describe the gut bacterial community associated to different larval stages of *A. fraterculus* sp. 1. This would provide a complete picture of the diversity and the potential function of gut bacteria associated to the larval stage in this species. Studies about the association between flies and their bacterial symbionts will surely improve our understanding about the biology of Tephritidae fruit fly pests and thus contribute to develop or improve sustainable control techniques (Deutscher et al., 2019; Raza et al., 2020).

## Data Availability Statement

The datasets presented in this study can be found in online repositories. The names of the repository/repositories and accession number(s) can be found in the article/Supplementary Material.

## Author Contributions

JS, SL, AD, GT, KB, AN, and DS conceived and designed the study. JS, DS, AN, FM, MV, GB, and LG conducted the sampling and identification of wild flies. JS, MR, EA, and PS carried out the DNA extraction and preparation of samples for sequencing. JS, EA, GT, PS, KB, and AD analyzed the results. JS, DS, SL, KB, AD, LG, and GT drafted the manuscript. All authors reviewed the manuscript.

## Conflict of Interest

The authors declare that the research was conducted in the absence of any commercial or financial relationships that could be construed as a potential conflict of interest.

## Publisher’s Note

All claims expressed in this article are solely those of the authors and do not necessarily represent those of their affiliated organizations, or those of the publisher, the editors and the reviewers. Any product that may be evaluated in this article, or claim that may be made by its manufacturer, is not guaranteed or endorsed by the publisher.
