# Supplementary material for: Analysis of the Gut Bacterial Community of Wild Larvae of Anastrepha fraterculus sp. 1: Effect of Host Fruit, Environment, and Prominent Stable Associations of the Genera Wolbachia, Tatumella, and Enterobacter
Source: Front Microbiol. 2022 Mar 10;13:822990. doi: 10.3389/fmicb.2022.822990 (PMC8960962; doi:10.3389/fmicb.2022.822990)
Supplement: Supplementary file 1 [file Data_Sheet_1.docx]

Supplementary Material

**Supplementary Table S1.** Percentage of samples where each OTU was detected and taxonomic description. Grey color indicates OTUs present in all groups (PC, PH, GC and GH); asterisk indicates OTUs detected in more than 50% of the total samples.

| OTU | **Phyllum** | **Class** | **Order** | **Family** | **Genus** | **%of**  **Total (S)** | **%of**  **PC(S)** | **%of PH(S)** | **%of GC(S)** | **%of GH(S)** |
| --- | --- | --- | --- | --- | --- | --- | --- | --- | --- | --- |
| OTU40***** | Bacteroidota | Bacteroidia | Chitinophagales | Chitinophagaceae | unk_  Chitinophagaceae | 63.33 | 23.33 | 33.33 | 96.67 | 100.00 |
| OTU26 | Bacteroidota | Bacteroidia | Flavobacteriales | Weeksellaceae | Chishuiella | 5.00 | 0.00 | 3.33 | 13.33 | 3.33 |
| OTU17***** | Cyanobacteria | Melainabacteria | Obscuribacterales | unk_  Cyanobacterium | unk_  Cyanobacterium | 82.50 | 53.33 | 76.67 | 100.00 | 100.00 |
| OTU23 | Firmicutes | Bacilli | Bacillales | Bacillaceae | Bacillus | 13.33 | 0.00 | 0.00 | 16.67 | 36.67 |
| OTU12 | Firmicutes | Bacilli | Lactobacillales | Lactobacillaceae | Lactobacillus | 4.17 | 0.00 | 0.00 | 10.00 | 6.67 |
| OTU20 | Firmicutes | Bacilli | Lactobacillales | Lactobacillaceae | Lactobacillus | 5.83 | 0.00 | 0.00 | 16.67 | 6.67 |
| OTU16 | Firmicutes | Bacilli | Lactobacillales | Leuconostocaceae | Leuconostoc | 18.33 | 33.33 | 16.67 | 10.00 | 13.33 |
| OTU27 | Firmicutes | Bacilli | Lactobacillales | Leuconostocaceae | Weissella | 6.67 | 13.33 | 3.33 | 10.00 | 0.00 |
| OTU7 | Firmicutes | Bacilli | Lactobacillales | Leuconostocaceae | Weissella | 9.17 | 33.33 | 3.33 | 0.00 | 0.00 |
| OTU10 | Proteobacteria | Alphaproteobacteria | Acetobacterales | Acetobacteraceae | Acetobacter | 28.33 | 53.33 | 6.67 | 23.33 | 30.00 |
| OTU199***** | Proteobacteria | Alphaproteobacteria | Acetobacterales | Acetobacteraceae | Gluconobacter | 85.00 | 86.67 | 73.33 | 93.33 | 86.67 |
| OTU391 | Proteobacteria | Alphaproteobacteria | Acetobacterales | Acetobacteraceae | Gluconobacter | 21.67 | 16.67 | 0.00 | 53.33 | 16.67 |
| OTU5 ***** | Proteobacteria | Alphaproteobacteria | Acetobacterales | Acetobacteraceae | Gluconobacter | 97.50 | 100.00 | 90.00 | 100.00 | 100.00 |
| OTU15 | Proteobacteria | Alphaproteobacteria | Acetobacterales | Acetobacteraceae | Komagataeibacter | 11.67 | 13.33 | 0.00 | 26.67 | 6.67 |
| OTU22***** | Proteobacteria | Alphaproteobacteria | Micropepsales | Micropepsaceae | unk_  Micropepsaceae | 83.33 | 70.00 | 63.33 | 100.00 | 100.00 |
| OTU24***** | Proteobacteria | Alphaproteobacteria | Rhizobiales | Xanthobacteraceae | Bradyrhizobium | 90.83 | 73.33 | 90.00 | 100.00 | 100.00 |
| OTU1***** | Proteobacteria | Alphaproteobacteria | Rickettsiales | Anaplasmataceae | Wolbachia | 100.00 | 100.00 | 100.00 | 100.00 | 100.00 |
| OTU75 | Proteobacteria | Gammaproteobacteria | Enterobacterales | Enterobacteriaceae | Buttiauxella | 4.17 | 0.00 | 6.67 | 0.00 | 10.00 |
| OTU4***** | Proteobacteria | Gammaproteobacteria | Enterobacterales | Enterobacteriaceae | Enterobacter | 100.00 | 100.00 | 100.00 | 100.00 | 100.00 |
| OTU654 | Proteobacteria | Gammaproteobacteria | Enterobacterales | Enterobacteriaceae | Enterobacter | 12.50 | 3.33 | 33.33 | 10.00 | 3.33 |
| OTU13***** | Proteobacteria | Gammaproteobacteria | Enterobacterales | Enterobacteriaceae | Escherichia-Shigella | 98.33 | 100.00 | 93.33 | 100.00 | 100.00 |
| OTU281 | Proteobacteria | Gammaproteobacteria | Enterobacterales | Enterobacteriaceae | Klebsiella | 7.50 | 0.00 | 6.67 | 10.00 | 13.33 |
| OTU109 | Proteobacteria | Gammaproteobacteria | Enterobacterales | Enterobacteriaceae | Kosakonia | 15.00 | 13.33 | 20.00 | 3.33 | 23.33 |
| OTU29 | Proteobacteria | Gammaproteobacteria | Enterobacterales | Enterobacteriaceae | Kosakonia | 5.00 | 0.00 | 3.33 | 6.67 | 10.00 |
| OTU302 | Proteobacteria | Gammaproteobacteria | Enterobacterales | Enterobacteriaceae | Pantoea | 8.33 | 3.33 | 30.00 | 0.00 | 0.00 |
| OTU614 | Proteobacteria | Gammaproteobacteria | Enterobacterales | Enterobacteriaceae | Pantoea | 4.17 | 0.00 | 10.00 | 6.67 | 0.00 |
| OTU9 | Proteobacteria | Gammaproteobacteria | Enterobacterales | Enterobacteriaceae | Pantoea | 17.50 | 10.00 | 13.33 | 16.67 | 30.00 |
| OTU38 | Proteobacteria | Gammaproteobacteria | Enterobacterales | Enterobacteriaceae | Pectobacterium | 3.33 | 3.33 | 3.33 | 3.33 | 3.33 |
| OTU51 | Proteobacteria | Gammaproteobacteria | Enterobacterales | Enterobacteriaceae | Sodalis | 19.17 | 20.00 | 13.33 | 26.67 | 16.67 |
| OTU19 | Proteobacteria | Gammaproteobacteria | Enterobacterales | Enterobacteriaceae | Tatumella | 7.50 | 0.00 | 13.33 | 6.67 | 10.00 |
| OTU245 | Proteobacteria | Gammaproteobacteria | Enterobacterales | Enterobacteriaceae | Tatumella | 7.50 | 13.33 | 3.33 | 3.33 | 10.00 |
| OTU3***** | Proteobacteria | Gammaproteobacteria | Enterobacterales | Enterobacteriaceae | Tatumella | 100.00 | 100.00 | 100.00 | 100.00 | 100.00 |
| OTU95***** | Proteobacteria | Gammaproteobacteria | Enterobacterales | Enterobacteriaceae | Tatumella | 15.83 | 6.67 | 6.67 | 20.00 | 30.00 |
| OTU18 | Proteobacteria | Gammaproteobacteria | Enterobacterales | unk_  Enterobacterales | unk_  Enterobacterales | 5.00 | 0.00 | 6.67 | 6.67 | 6.67 |
| OTU32 | Proteobacteria | Gammaproteobacteria | Enterobacterales | Enterobacteriaceae | Raoultella | 14.17 | 13.33 | 26.67 | 6.67 | 10.00 |
| OTU319 | Proteobacteria | Gammaproteobacteria | Enterobacterales | unk_  Enterobacterales | unk_  Enterobacterales | 14.17 | 6.67 | 3.33 | 20.00 | 26.67 |
| OTU42 | Proteobacteria | Gammaproteobacteria | Orbales | Orbaceae | unk_  Orbaceae | 7.50 | 20.00 | 3.33 | 0.00 | 6.67 |
| OTU11 | Proteobacteria | Gammaproteobacteria | Pseudomonadales | Pseudomonadaceae | Pseudomonas | 3.33 | 0.00 | 10.00 | 0.00 | 3.33 |
| OTU21 | Proteobacteria | Gammaproteobacteria | Pseudomonadales | Pseudomonadaceae | Pseudomonas | 6.67 | 0.00 | 26.67 | 0.00 | 0.00 |
| OTU8***** | Proteobacteria | Gammaproteobacteria | Xanthomonadales | Rhodanobacteraceae | Frateuria | 89.17 | 93.33 | 80.00 | 93.33 | 90.00 |

**Supplementary Table S2**. Percentage of reads assigned per OTU and taxonomic description. Grey color indicates OTUs present in all groups (PC, PH, GC and GH); asterisk indicates OTUs that represent more than 2% of total reads.

| OTU | **Phyllum** | **Class** | **Order** | **Family** | **Genus** | **%of total(R)** | **%of PC(R)** | **%of PH(R)** | **%of GC(R)** | **%of GH(R)** |
| --- | --- | --- | --- | --- | --- | --- | --- | --- | --- | --- |
| OTU40 | Bacteroidota | Bacteroidia | Chitinophagales | Chitinophagaceae | unk_ Chitinophagaceae | 0.117 | 0.004 | 0.004 | 0.226 | 0.269 |
| OTU26 | Bacteroidota | Bacteroidia | Flavobacteriales | Weeksellaceae | Chishuiella | 0.189 | 0.000 | 0.006 | 0.777 | 0.029 |
| OTU17 | Cyanobacteria | Melainabacteria | Obscuribacterales | unk_  Cyanobacterium | unk_  Cyanobacterium | 0.261 | 0.009 | 0.018 | 0.552 | 0.546 |
| OTU23 | Firmicutes | Bacilli | Bacillales | Bacillaceae | Bacillus | 0.196 | 0.000 | 0.000 | 0.470 | 0.376 |
| OTU12 | Firmicutes | Bacilli | Lactobacillales | Lactobacillaceae | Lactobacillus | 1.065 | 0.000 | 0.000 | 4.485 | 0.102 |
| OTU20 | Firmicutes | Bacilli | Lactobacillales | Lactobacillaceae | Lactobacillus | 0.387 | 0.000 | 0.000 | 1.637 | 0.031 |
| OTU16 | Firmicutes | Bacilli | Lactobacillales | Leuconostocaceae | Leuconostoc | 0.454 | 0.659 | 0.301 | 0.514 | 0.481 |
| OTU27 | Firmicutes | Bacilli | Lactobacillales | Leuconostocaceae | Weissella | 0.195 | 0.732 | 0.021 | 0.088 | 0.000 |
| OTU7 | Firmicutes | Bacilli | Lactobacillales | Leuconostocaceae | Weissella | 2.881 | 10.925 | 1.486 | 0.000 | 0.000 |
| OTU10***** | Proteobacteria | Alphaproteobacteria | Acetobacterales | Acetobacteraceae | Acetobacter | 5.402 | 13.433 | 0.618 | 3.705 | 5.512 |
| OTU199 | Proteobacteria | Alphaproteobacteria | Acetobacterales | Acetobacteraceae | Gluconobacter | 1.946 | 1.335 | 0.512 | 1.656 | 4.880 |
| OTU391 | Proteobacteria | Alphaproteobacteria | Acetobacterales | Acetobacteraceae | Gluconobacter | 0.348 | 0.188 | 0.000 | 0.968 | 0.342 |
| OTU5***** | Proteobacteria | Alphaproteobacteria | Acetobacterales | Acetobacteraceae | Gluconobacter | 6.246 | 4.659 | 3.856 | 8.127 | 10.260 |
| OTU15 | Proteobacteria | Alphaproteobacteria | Acetobacterales | Acetobacteraceae | Komagataeibacter | 1.374 | 0.272 | 0.000 | 5.151 | 0.494 |
| OTU22 | Proteobacteria | Alphaproteobacteria | Micropepsales | Micropepsaceae | unk_ Micropepsaceae | 0.157 | 0.014 | 0.014 | 0.260 | 0.387 |
| OTU24 | Proteobacteria | Alphaproteobacteria | Rhizobiales | Xanthobacteraceae | Bradyrhizobium | 0.158 | 0.031 | 0.020 | 0.271 | 0.360 |
| OTU1***** | Proteobacteria | Alphaproteobacteria | Rickettsiales | Anaplasmataceae | Wolbachia | 33.545 | 57.826 | 55.711 | 17.053 | 13.895 |
| OTU75 | Proteobacteria | Gammaproteobacteria | Enterobacterales | Enterobacteriaceae | Buttiauxella | 0.253 | 0.000 | 0.138 | 0.000 | 0.953 |
| OTU4***** | Proteobacteria | Gammaproteobacteria | Enterobacterales | Enterobacteriaceae | Enterobacter | 11.134 | 6.129 | 13.505 | 15.692 | 12.632 |
| OTU654 | Proteobacteria | Gammaproteobacteria | Enterobacterales | Enterobacteriaceae | Enterobacter | 0.709 | 0.016 | 2.956 | 0.067 | 0.014 |
| OTU13 | Proteobacteria | Gammaproteobacteria | Enterobacterales | Enterobacteriaceae | Escherichia-Shigella | 0.771 | 0.669 | 0.624 | 0.922 | 1.105 |
| OTU281 | Proteobacteria | Gammaproteobacteria | Enterobacterales | Enterobacteriaceae | Klebsiella | 1.011 | 0.000 | 2.229 | 0.257 | 1.868 |
| OTU109 | Proteobacteria | Gammaproteobacteria | Enterobacterales | Enterobacteriaceae | Kosakonia | 1.325 | 2.543 | 1.897 | 0.041 | 1.225 |
| OTU29 | Proteobacteria | Gammaproteobacteria | Enterobacterales | Enterobacteriaceae | Kosakonia | 0.219 | 0.000 | 0.009 | 0.311 | 0.623 |
| OTU302 | Proteobacteria | Gammaproteobacteria | Enterobacterales | Enterobacteriaceae | Pantoea | 0.285 | 0.019 | 1.209 | 0.000 | 0.000 |
| OTU614 | Proteobacteria | Gammaproteobacteria | Enterobacterales | Enterobacteriaceae | Pantoea | 0.353 | 0.000 | 0.129 | 1.393 | 0.000 |
| OTU9***** | Proteobacteria | Gammaproteobacteria | Enterobacterales | Enterobacteriaceae | Pantoea | 3.847 | 1.295 | 7.999 | 1.678 | 5.597 |
| OTU38 | Proteobacteria | Gammaproteobacteria | Enterobacterales | Enterobacteriaceae | Pectobacterium | 0.288 | 0.010 | 1.172 | 0.028 | 0.031 |
| OTU51 | Proteobacteria | Gammaproteobacteria | Enterobacterales | Enterobacteriaceae | Sodalis | 0.144 | 0.053 | 0.375 | 0.084 | 0.110 |
| OTU19 | Proteobacteria | Gammaproteobacteria | Enterobacterales | Enterobacteriaceae | Tatumella | 0.172 | 0.000 | 0.540 | 0.058 | 0.141 |
| OTU245 | Proteobacteria | Gammaproteobacteria | Enterobacterales | Enterobacteriaceae | Tatumella | 0.678 | 1.359 | 0.716 | 0.030 | 0.816 |
| OTU3***** | Proteobacteria | Gammaproteobacteria | Enterobacterales | Enterobacteriaceae | Tatumella | 16.855 | 9.114 | 4.061 | 25.559 | 33.866 |
| OTU95 | Proteobacteria | Gammaproteobacteria | Enterobacterales | Enterobacteriaceae | Tatumella | 0.461 | 0.062 | 0.149 | 1.236 | 0.540 |
| OTU18 | Proteobacteria | Gammaproteobacteria | Enterobacterales | unk_ Enterobacterales | unk_ Enterobacterales | 0.785 | 0.000 | 0.122 | 0.144 | 3.113 |
| OTU32 | Proteobacteria | Gammaproteobacteria | Enterobacterales | Enterobacteriaceae | Raoultella | 1.353 | 0.833 | 4.235 | 0.577 | 0.181 |
| OTU319 | Proteobacteria | Gammaproteobacteria | Enterobacterales | unk_ Enterobacterales | unk_ Enterobacterales | 1.082 | 0.211 | 0.176 | 2.843 | 1.431 |
| OTU42 | Proteobacteria | Gammaproteobacteria | Orbales | Orbaceae | unk_ Orbaceae | 0.140 | 0.464 | 0.125 | 0.000 | 0.016 |
| OTU11 | Proteobacteria | Gammaproteobacteria | Pseudomonadales | Pseudomonadaceae | Pseudomonas | 0.764 | 0.000 | 3.254 | 0.000 | 0.035 |
| OTU21 | Proteobacteria | Gammaproteobacteria | Pseudomonadales | Pseudomonadaceae | Pseudomonas | 0.168 | 0.000 | 0.725 | 0.000 | 0.000 |
| OTU8***** | Proteobacteria | Gammaproteobacteria | Xanthomonadales | Rhodanobacteraceae | Frateuria | 2.280 | 2.017 | 2.765 | 3.139 | 1.899 |


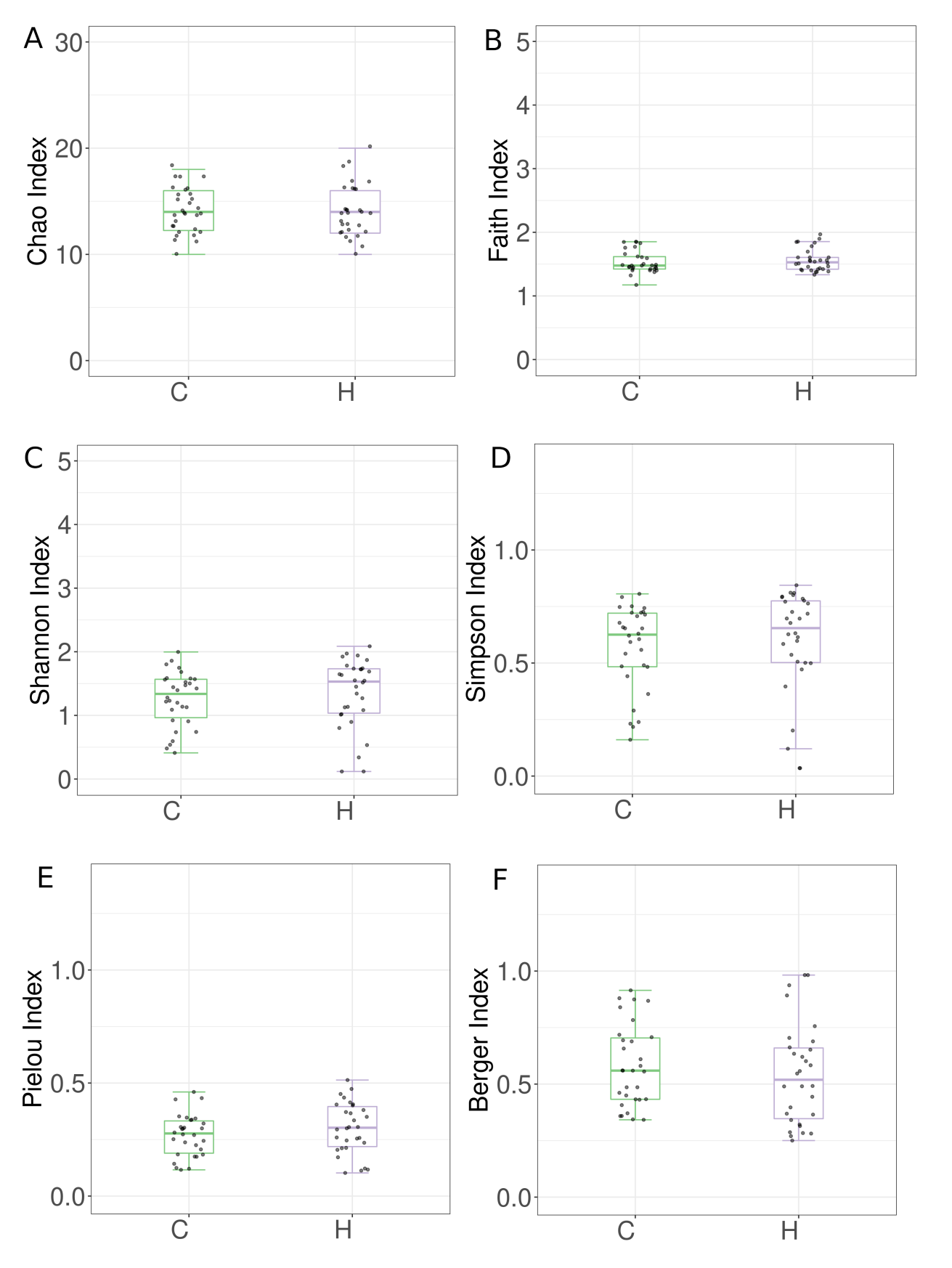


**Supplementary Figure S1**. α-Diversity analyses of larval gut bacterial community associated to wild Anastrepha fraterculus. The figure presents the comparison between larvae collected exclusively from guava fruits from different origins HorcoMolle (H) and Concordia (C). A) Chao index; B) Faith index; C) Shannon index; D) Simpson Index; E) Pielou Index; F) Berger Index. Dots indicate observed values and box plots depict means and standard deviation. Kruskal–Wallis Rank Sum test p-values are plotted for each paired comparison.


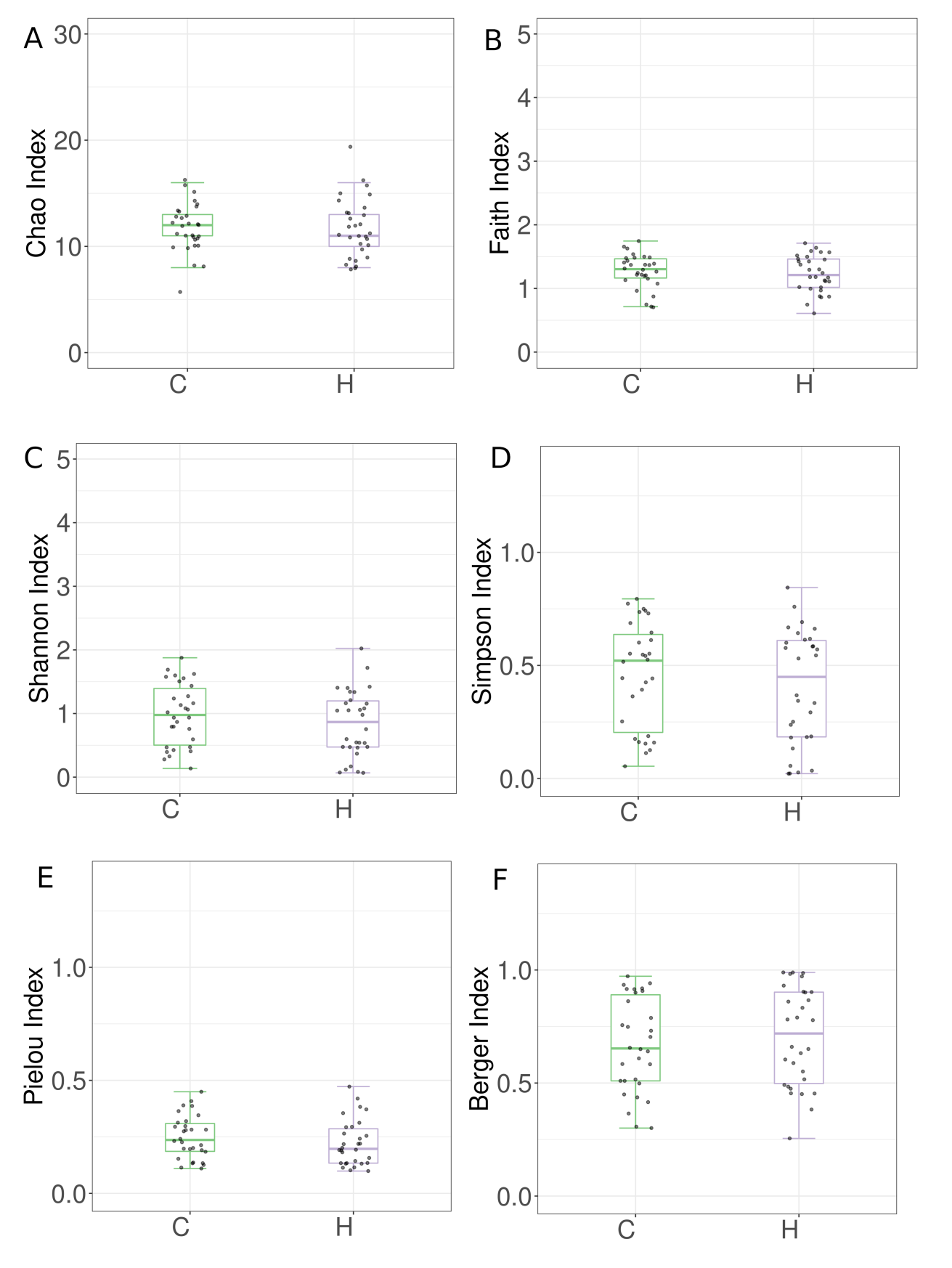


**Supplementary Figure S2**. α-Diversity analyses of larval gut bacterial community associated to wild Anastrepha fraterculus. The figure presents the comparison between larvae collected exclusively from peach fruits from different origins HorcoMolle (H) and Concordia (C). A) Chao index; B) Faith index; C) Shannon index; D) Simpson Index; E) Pielou Index; F) Berger Index. Dots indicate observed values and box plots depict means and standard deviation. Kruskal–Wallis Rank Sum test p-values are plotted for each paired comparison
